# Phylogeographical Landscape of Citrobacter portucalensis Carrying Clinically Relevant Resistomes

**DOI:** 10.1128/spectrum.01506-21

**Published:** 2022-03-31

**Authors:** Fábio P. Sellera, Miriam R. Fernandes, Bruna Fuga, Herrison Fontana, Felipe Vásquez-Ponce, Daphne W. Goldberg, Daniel F. Monte, Larissa Rodrigues, Adriana R. Cardenas-Arias, Ralf Lopes, Brenda Cardoso, Daniela G. C. Costa, Fernanda Esposito, Nilton Lincopan

**Affiliations:** a Department of Internal Medicine, School of Veterinary Medicine and Animal Science, University of São Paulo, São Paulo, Brazil; b One Health Brazilian Resistance Project (OneBR), São Paulo, Brazil; c School of Veterinary Medicine, Metropolitan University of Santos, Santos, Brazil; d Department of Clinical Analysis, School of Pharmacy, University of São Paulo, São Paulo, Brazil; e Department of Microbiology, Instituto de Ciências Biomédicas, Universidade de São Paulo, São Paulo, Brazil; f Aquatic Biodiversity Monitoring Program, Federal University of Rio Grande, Rio Grande do Sul, Brazil; g Department of Food and Experimental Nutrition, Faculty of Pharmaceutical Sciences, Food Research Center, University of São Paulo, São Paulo, Brazil; h Pró-Tamar Foundation, Ubatuba, São Paulo, Brazil; The National University of Singapore and the Genome Institute of Singapore

**Keywords:** Enterobacterales, emerging pathogens, multidrug-resistant, international clade, critical priority, One Health, genomic surveillance

## Abstract

During a surveillance study conducted to assess the occurrence and genomic landscape of critical priority pathogens circulating at the human-animal-environment interface in Brazil, as part of the Grand Challenges Explorations-New Approaches to Characterize the Global Burden of Antimicrobial Resistance program, two multidrug-resistant (MDR) Citrobacter portucalensis carrying *bla*_CTX-M-15_ extended-spectrum β-lactamase (ESBL) genes, isolated from green sea turtles, were characterized. Genomic and phylogeographical analysis of C. portucalensis genomes available in public databases revealed the intercontinental dissemination of clades carrying different arrays of clinically relevant genes conferring resistance to carbapenems, broad-spectrum cephalosporins, cephamycins, aminoglycosides and fluoroquinolones, disinfectants, and heavy metals. Our observations suggest that C. portucalensis could be emerging as critical priority bacteria of both public and One Health importance worldwide.

**IMPORTANCE** The global spread of antibiotic-resistant priority pathogens beyond the hospital setting is a critical issue within a One Health context that integrates the human-animal-environment interfaces. On the other hand, next-generation sequencing technologies along with user-friendly and high-quality bioinformatics tools have improved the identification of bacterial species, and bacterial resistance surveillance. The novel Citrobacter portucalensis species was proposed in 2017 after taxonomic reclassification and definition of the strain A60^T^ isolated in 2008. Here, we presented genomic data showing the occurrence of multidrug-resistant C. portucalensis isolates carrying *bla*_CTX-M-15_ ESBL genes in South America. Additionally, we observed the intercontinental dissemination of clades harboring a broad resistome to clinically relevant antibiotics. Therefore, these findings highlight that C. portucalensis is a global MDR bacteria that carries intrinsic *bla*_CMY_- and *qnrB*-type genes and has become a critical priority pathogen due to the acquisition of clinically relevant resistance determinants, such as ESBL and carbapenemase-encoding genes.

## OBSERVATION

Members of the genus *Citrobacter* are part of the normal intestinal flora of humans and animals and have been isolated from a variety of environmental sources, including soil, water, and food; being considered opportunistic pathogens for humans (1, 2). The clinical significance of *Citrobacter* is due to its ability to easily acquire multiple resistance genes to antibiotics used in therapy. In this regard, some species of the genus carry intrinsic AmpC-type β-lactamase genes (i.e., *bla*_CMY_-type), which can be related to mobile genetic elements (1, 3). Additionally, ESBLs and carbapenemases have been identified in *Citrobacter* spp., expanding the resistance to broad-spectrum cephalosporins and carbapenems. Currently, this genus comprises 15 species (https://lpsn.dsmz.de/genus/citrobacter) with the most recently described *Citrobacter* species being C. portucalensis, C. europaeus, and C. cronae (1, 4, 5). Although the novel C. portucalensis species was proposed in 2017, the strain A60^T^ was isolated in 2008 from a water well sample collected in Cantanhede city, Centre region of Portugal (1, 2). Consequently, by using genome data of C. portucalensis A60^T^, the average nucleotide identity (ANI) results have led to change originally genomes submitted as C. freundii and *Citrobacter* spp., to C. portucalensis since 2018.

Antibiotic-resistant priority pathogens have globally disseminated beyond the hospital environment, threatening wildlife and ecosystems within a One Health context that integrates the human-animal-environment interface (6–8). During a nationwide surveillance study (One Health Brazilian Resistance Program, OneBR), conducted in Brazil to assess the occurrence and genomic features of World Health Organization (WHO) priority pathogens circulating at the human-animal-environment interface (6), two ceftriaxone-resistant Gram-negative bacilli isolated from cloacal swab samples collected from 2 green sea turtles affected by fibropapillomatosis (a debilitating neoplastic disease with immunosuppressive effects), were sequenced. Both green sea turtles were admitted to a rescue and rehabilitation center in Southeastern Brazil (23°27'08.0"S, 45°04'13.6"W). Isolates were recovered from MacConkey agar plates supplemented with ceftriaxone (2 μg/mL) and incubated at 37°C overnight. Bacterial identification and antimicrobial susceptibility testing were performed by Vitek 2 system (bioMérieux, USA), disc diffusion, Etest, and/or agar dilution methods (9). Colistin MIC was determined by broth microdilution method, according to EUCAST (http://www.eucast.org). Extended-spectrum β-lactamase (ESBL) production was screened by the double-disk synergy test (DDST) (10).

For whole-genome sequencing (WGS), genomic DNAs were extracted using the PureLink Quick Gel Extraction kit (Life Technologies, Carlsbad, CA). DNA concentrations were evaluated using a Qubit 2.0 fluorometer (Life Technologies, Carlsbad, CA). Genomic libraries were constructed using a Nextera XT DNA library preparation kit (Illumina Inc., Cambridge, UK) and genomic DNA was sequenced by the Illumina NextSeq 500 platform, using paired-end reads (150 bp). Short-read sequence data were *de novo* assembled using CLC Genomics Workbench v.10. Unassembled reads and contigs less than 200-bp long were removed from the genome. The sequences were annotated using PGAP v.3.2 (http://www.ncbi.nlm.nih.gov/genome/annotation_prok/), and ANI was based on the NCBI report (https://www.ncbi.nlm.nih.gov/) and by using DFAST (https://dfast.ddbj.nig.ac.jp/). Resistome and plasmidome were evaluated using ResFinder 4.1 and PlasmidFinder 2.1 databases (http://www.genomicepidemiology.org/). Disinfectants (quaternary ammonium compounds [QACs]), pesticides, and heavy metal resistance genes were predicted using ABRicate (https://github.com/tseemann/abricate) and comparing the contigs against the NCBI database (https://www.ncbi.nlm.nih.gov). Genes were predicted using a coverage and identity threshold > 80%. Sequence types were predicted using the *Citrobacter* spp. Multilocus sequence type (MLST) scheme (https://pubmlst.org/organisms/citrobacter-spp). Phylogenomic analysis was performed using publicly available genomic data (https://www.ncbi.nlm.nih.gov) from 69 C. portucalensis strains isolated from different sources and countries (Table S1). In this regard, CSI Phylogeny v.1.4 (https://cge.cbs.dtu.dk/) was used for SNP-based phylogenetic tree inference using the C. portucalensis FDAARGOS_617 complete chromosome sequence (GenBank accession number: CP044098.1) as reference. Tree topology visualization and annotation were performed with iTol v.6 (https://itol.embl.de/).

Initially, cephalosporin-resistant isolates TV06 and TV13 were identified as *Citrobacter* spp. by Vitek 2 system, being further investigated by WGS. In this regard, genomes of TV06 and TV13 strains were 5,357,700-bp and 5,414,211-bp in size with 126 and 113 of average coverage and 51.98% and 51.54% GC content, respectively; and both presented an average read length of 150-bp. Additionally, genome sequences of TV06 (GenBank accession number: VTZD01000001.1) and TV13 (GenBank accession number: VTZC01000001.1) strains shared 98.37% and 98.53% average nucleotide identity with the genome of C. portucalensis strain A60^T^ (GenBank accession number: MVFY01000001.1), respectively. Thus, these sequences were assigned to the C. portucalensis species by the NCBI taxonomy staff.

Both strains exhibited a multidrug-resistant profile (i.e., resistance to at least one agent in three or more antimicrobial categories) (11), against amoxicillin-clavulanic acid, aztreonam, ceftriaxone (MIC > 32 μg/mL), cefotaxime, ceftazidime (MIC ≥ 64 μg/mL), cefepime (MIC ≥ 32 μg/mL), cefoxitin (MIC ≥ 64 μg/mL), gentamicin (MIC ≥ 64 μg/mL), nalidixic acid, ciprofloxacin (MIC ≥ 4 μg/mL), and trimethoprim-sulfamethoxazole (≥320, 16/304). On the other hand, both strains showed an ESBL phenotype, remaining susceptible to carbapenems (ertapenem, MIC ≤ 0.12 μg/mL; imipenem, MIC ≤ 0.25 μg/mL; and meropenem, MIC ≤ 0.25 μg/mL), fosfomycin (MIC ≤ 4 μg/mL), and colistin (MIC = 0.5 μg/mL).

Results from WGS analysis are summarized in Table S1. The resistome of both C. portucalensis strains was composed of genes conferring resistance to β-lactams (*bla*_CTX-M-15_, *bla*_CMY-129_-like, *bla*_TEM-1B_, *bla*_OXA-1_), aminoglycosides, quinolones, sulfonamides, and trimethoprim. C. portucalensis TV06 harbored additional resistance genes to phenicols, fosfomycin, and tetracyclines. On the other hand, genes conferring resistance to heavy metals (arsenic, mercury, silver, and tellurium), disinfectants (quaternary ammonium compounds [QACs]), and pesticides (glyphosate) were detected in both genomes (Table S1). Plasmid analysis revealed the presence of IncHI2 in C. portucalensis TV06, and IncHI1, IncFII, and IncFIB plasmids in the strain TV13. In this regard, *in silico* analysis mapping the contigs against paired-end short reads along to BLASTn analysis revealed that *bla*_CTX-M-15_ genes were carried by IncHI and IncFII plasmids in C. portucalensis TV06 and TV13, respectively (Table S1). Unfortunately, because we used short-read sequencing technology, it was not possible to assemble the complete plasmids.

**FIG 1:**
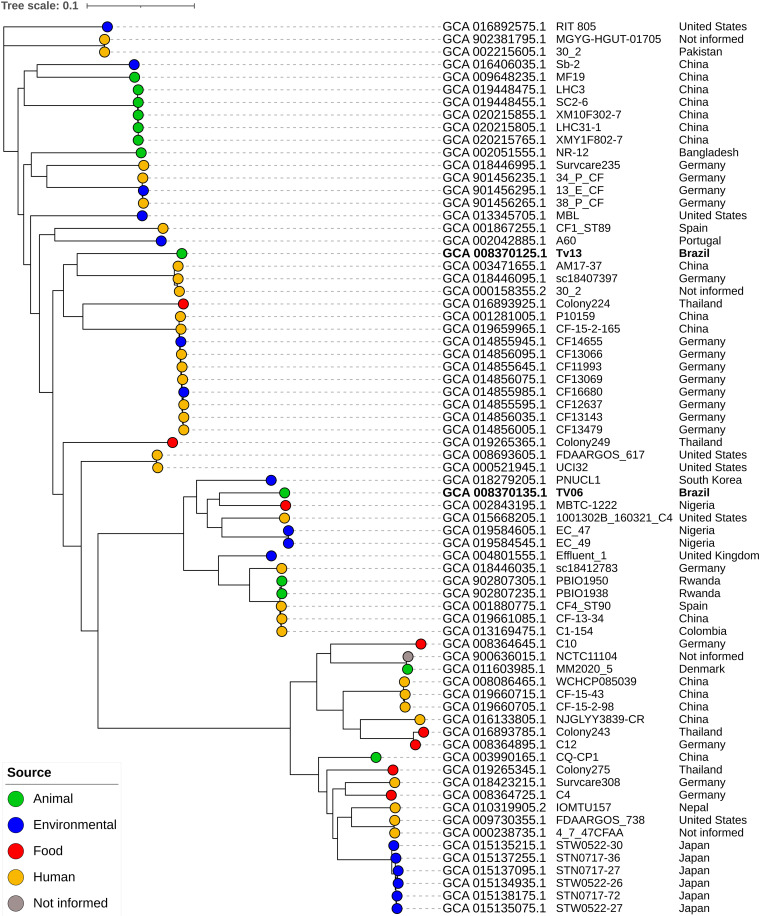
SNP-based phylogenetic analysis of CTX-M-15-positive Citrobacter portucalensis strains TV06 (GenBank accession number: VTZD01000001.1) and TV13 (GenBank accession number: VTZC01000001.1) identified in this study, and 69 previously sequenced, assembled, and annotated C. portucalensis human and nonhuman C. portucalensis strains identified in European, Asian, North American, South American, and African countries (Table S1). Phylogenetic relationships were inferred using CSI Phylogeny 1.4 (https://cge.cbs.dtu.dk/). The C. portucalensis FDAARGOS_617 complete chromosome sequence (GenBank accession number: CP044098.1) was used as the reference genome. The phylogenetic tree was visualized by using iTOL v.6 (https://itol.embl.de/). For each C. portucalensis isolate the assembly accession number, strain name, and the country is quoted. The scale bar refers to branch lengths and indicates the number of substitutions per site based on 3303375 nucleotide positions (67.04% of the reference genome covered by all isolates) found in all analyzed genomes, using 10 bp as the minimum distance between SNPs. The minimum and maximum SNP differences among all C. portucalensis isolates were 0 and 40262, respectively.

Although, MLST analysis revealed that the TV13 strain belonged to ST63, it was not possible to predict the ST of the TV06 strain. SNP-based phylogenetic analysis grouped CTX-M-15-positive TV06 and TV13 strains in two distinct clades, both related to C. portucalensis strains isolated from human and nonhuman sources in distinct geographic regions (Fig. 1). The matrix of SNP-based phylogeny analysis is quoted in Table S2. Specifically, the TV06 strain clustered (14549 to 15041 SNP differences) with C. portucalensis strains MBTC-1222 (isolated from food in Nigeria; assembly accession number: GCA_002843195.2), 1001302B_160321_C4 (isolated from a human in the USA; assembly accession number: GCA_015668205.1), and EC_47 and EC_49 (isolated from environmental sources in Nigeria; Assembly accession numbers: GCA_019584605.1 and GCA_019584545.1) (Fig. 1, Table S2). On the other hand, TV13 was clustered (21254 to 21829 SNP differences) with the human strains AM17-37 and sc18407397, identified in China and Germany, respectively (assembly accession numbers: GCA_003471655.1 and GCA_018446095.1); the food strain Colony224 from Thailand (GCA_016893925.1); human strains P10159 and CF-15-2-165 from China (Assembly accession numbers: GCA_001281005.1 and GCA_019659965.1); and CF14655, CF13066, CF11993, CF13069, CF16680, CF12637, CF13143 and CF13479 (Assembly accession numbers: GCA_014855945.1, GCA_014856095.1, GCA_014855645.1, GCA_014856075.1, GCA_014855985.1, GCA_014855595.1, GCA_014856035.1, and GCA_014856005.1) strains isolated from human and environmental sources in Germany.

**FIG 2:**
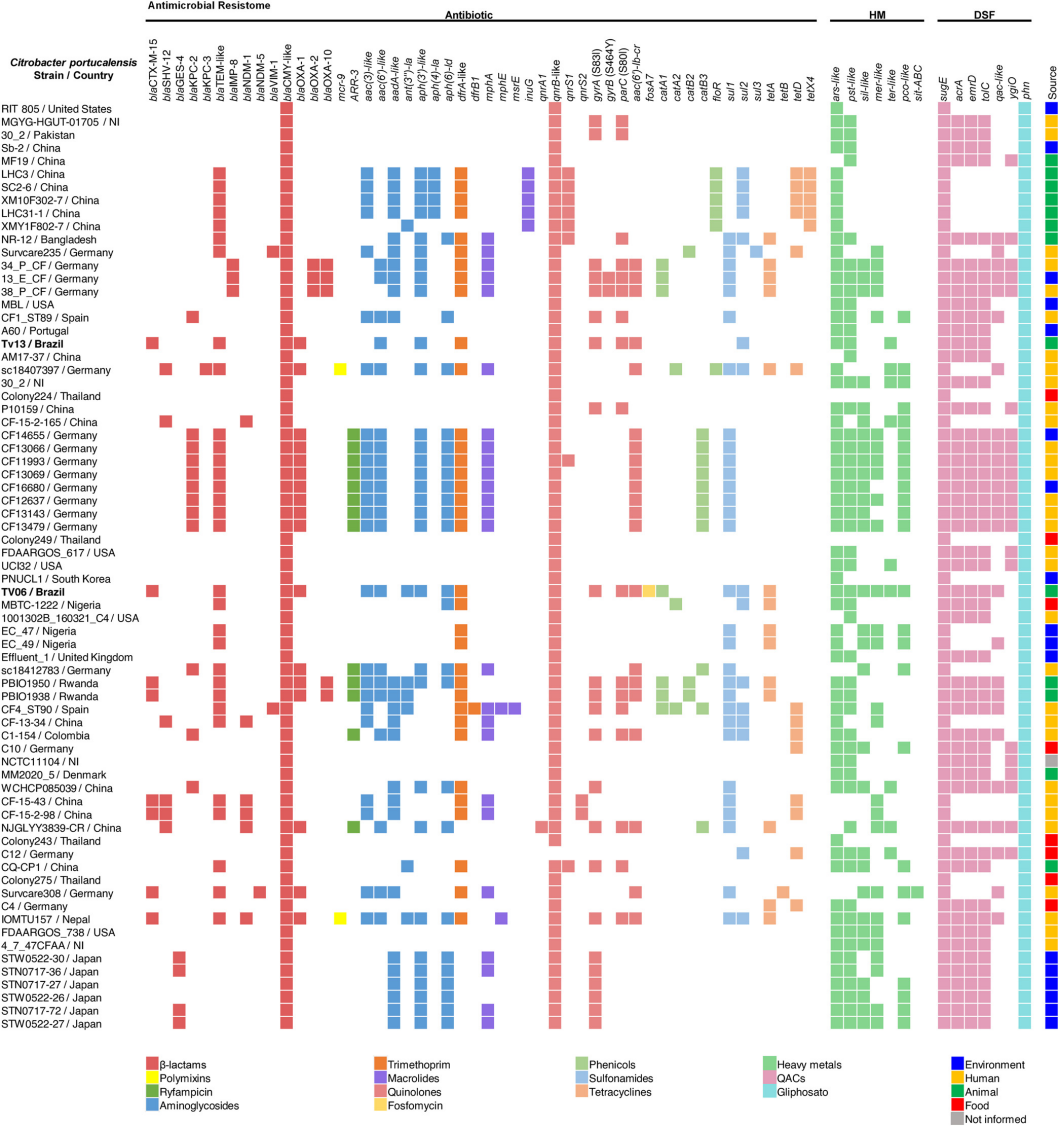
Heatmap showing the antimicrobial resistome of Citrobacter portucalensis strains isolated from human and nonhuman sources worldwide (Table S1). HM, heavy metal resistome. DSF, disinfectant resistome. NI, not informed.

Remarkably, C. portucalensis global strains harbor genes encoding resistance to critically important antimicrobials, including carbapenems (*bla*_VIM-1_, *bla*_NDM-1_, *bla*_NDM-5_, *bla*_IMP-8_, *bla*_GES-4_, *bla*_KPC-2_ and *bla*_KPC-3_), broad-spectrum cephalosporins (*bla*_CTX-M-15_, *bla*_SHV-12_, and *bla*_OXA-_like), cephamycins (intrinsic *bla*_CMY-2_-, *bla*_CMY-4_-, *bla*_CMY-13_-, *bla*_CMY-25_-, *bla*_CMY-26_-, *bla*_CMY-34_-, *bla*_CMY-35_-, *bla*_CMY-39_-, *bla*_CMY-46_-, *bla*_CMY-49_-, *bla*_CMY-53_-, *bla*_CMY-63_-, *bla*_CMY-71_-, *bla*_CMY-86_-, *bla*_CMY-124_-, *bla*_CMY-127_- and *bla*_CMY-129_-like), quinolones (*qnrA1, qnrB1, qnrB2, qnrB6, qnrB9, qnrB13, qnrB15, qnrB17, qnr18, qnrB19, qnrB23, qnrB29, qnrB30, qnrB32, qnrB54, qnrB57, qnrB58, qnrB76, qnrB77, qnrS1, qnrS2, and/or aac[6’]Ib-cr*), polymyxins (*mcr-9*), and aminoglycosides (Fig. 2, Table S1).

Strikingly, we identified that both TV06 and TV13 isolates harbored the clinically relevant *bla*_CTX-M-15_ gene, which confers resistance to expanded spectrum cephalosporins, being on the spotlights due to its rapid spread among clinical and environmental members of Enterobacterales (12, 13). CTX-M-15 producers have gained medical attention as important pathogens responsible for life-threatening infections, being frequently associated with MDR profiles (12, 13). Noteworthy, we have recently identified CTX-M-15-producing Enterobacter hormaechei and C. freundii coinfecting a free-living green turtle (C. mydas) (14), supporting that CTX-M-15 producers also represent a threat for wildlife.

After having your updated name formally recognized, C. portucalensis has been further isolated from uziza leaves in Nigeria (15), nasal and urine samples in dogs from Japan (3), urinary tract infection in a cat from Austria (16), poultry dropping samples from Bangladesh (17), soil from USA (18), and human stool and sputum samples in China (19, 20). Moreover, from genome data deposited in the NCBI database, vegetables, humans, animals, and the environment have been registered as sources of isolation of C. portucalensis in Bangladesh, China, Colombia, Denmark, Germany, Japan, Nepal, Nigeria, Pakistan, Portugal, Rwanda, South Korea, Spain, Thailand, United Kingdom and USA (Table S1).

In this study, we report the emergence of CTX-M-15-producing C. portucalensis in marine wildlife, in South America. In this regard, green turtles (C. mydas) are endangered marine animals that have a global distribution, occurring throughout tropical and subtropical waters (https://www.iucnredlist.org/species/4615/11037468). Due to their highly migratory behavior, green turtles have been investigated as bioindicators of polluted ecosystems contaminated with MDR bacteria (21). Therefore, the occurrence of this sort of bacteria in natural environments can have serious ecological implications (22), adding another layer of difficulty to marine wildlife conservation strategies. On the other hand, considering that C. portucalensis is closely related to C. freundii (an important nosocomial pathogen) (1), continuous surveillance is required to provide more information about the global dissemination of this emergent species and its adaptation to different hosts.

In summary, our findings highlight that C. portucalensis is a global MDR pathogen with intrinsic genes encoding AmpC enzymes active on cephamycin (CMY), which has become a critical priority specie due to the acquisition of clinically significant resistance determinants, such as plasmid-mediated *bla*_CTX-M_-type ESBL and carbapenemase-encoding genes (i.e., *bla*_GES_, *bla*_VIM_, *bla*_NDM_, *bla*_IMP_, and *bla*_KPC_). Finally, because marine animals have been overlooked in the epidemiology of critically important pathogens, more research focusing on the transmission pathways of ESBL-producing bacteria in marine environments is required to understand the clinical and epidemiological impacts on their populations.

## References

[1] Ribeiro TG, Gonçalves BR, da Silva MS, Novais Â, Machado E, Carriço JA, Peixe L. 2017. *Citrobacter portucalensis* sp. nov., isolated from an aquatic sample. Int J Syst Evol Microbiol 67:3513–3517. doi:10.1099/ijsem.0.002154. 28857032

[2] Cho GS, Stein M, Bockelmann W, Neve H, Brinks E, Franz CMAP. 2018. Draft genome sequence of *Citrobacter gillenii* MBT-C3, isolated from lamb's lettuce. Microbiol Resour Announc 7:e01177-18. doi:10.1128/MRA.01177-18. 30533725 PMC6256432

[3] Harada K, Shimizu T, Ozaki H, Kimura Y, Miyamoto T, Tsuyuki Y. 2019. Characterization of antimicrobial resistance in *Serratia* spp. and *Citrobacter* spp. isolates from companion animals in Japan: nosocomial dissemination of extended-spectrum cephalosporin-resistant *Citrobacter freundii*. Microorganisms 7:64. doi:10.3390/microorganisms7030064. 30823419 PMC6462910

[4] Ribeiro TG, Clermont D, Branquinho R, Machado E, Peixe L, Brisse S. 2017. *Citrobacter europaeus* sp. nov., isolated from water and human faecal samples. Int J Syst Evol Microbiol 67:170–173. doi:10.1099/ijsem.0.001606. 27902229

[5] Oberhettinger P, Schule L, Marschal M, Bezdan D, Ossowski S, Dorfel D, Vogel W, Rossen JW, Willmann M, Peter S. 2020. Description of *Citrobacter cronae* sp. nov., isolated from human rectal swabs and stool samples. Int J Syst Evol Microbiol 70:2998–3003. doi:10.1099/ijsem.0.004100. 32375941 PMC7395625

[6] Tacconelli E, Carrara E, Savoldi A, Harbarth S, Mendelson M, Monnet DL, Pulcini C, Kahlmeter G, Kluytmans J, Carmeli Y, Ouellette M, Outterson K, Patel J, Cavaleri M, Cox EM, Houchens CR, Grayson ML, Hansen P, Singh N, Theuretzbacher U, Magrini N, WHO Pathogens Priority List Working Group. 2018. Discovery, research, and development of new antibiotics: the WHO priority list of antibiotic-resistant bacteria and tuberculosis. Lancet Infect Dis 18:318–327. doi:10.1016/S1473-3099(17)30753-3. 29276051

[7] Fuentes-Castillo D, Navas-Suárez PE, Gondim MF, Esposito F, Sacristán C, Fontana H, Fuga B, Piovani C, Kooij R, Lincopan N, Catão-Dias JL. 2021. Genomic characterization of multidrug-resistant ESBL-producing *Escherichia coli* ST58 causing fatal colibacillosis in critically endangered Brazilian merganser (*Mergus octosetaceus*). Transbound Emerg Dis 68:258–266. doi:10.1111/tbed.13686. 32544292 PMC8246901

[8] Hernando-Amado S, Coque TM, Baquero F, Martínez JL. 2020. Antibiotic resistance: moving from individual health norms to social norms in one health and global health. Front Microbiol 11:1914. doi:10.3389/fmicb.2020.01914. 32983000 PMC7483582

[9] Clinical and Laboratory Standards Institute. 2021. Performance Standards for Antimicrobial Susceptibility Testing. 31st ed CLSI supplement M100. CLSI, Wayne, PA, USA.

[10] Jarlier V, Nicolas MH, Fournier G, Philippon A. 1988. Extended broad-spectrum β lactamases conferring transferable resistance to newer beta-lactam agents in *Enterobacteriaceae*: hospital prevalence and susceptibility patterns. Rev Infect Dis 10:867–878. PMID: 3263690. doi:10.1093/clinids/10.4.867. 3263690

[11] Magiorakos AP, Srinivasan A, Carey RB, Carmeli Y, Falagas ME, Giske CG, Harbarth S, Hindler JF, Kahlmeter G, Olsson-Liljequist B, Paterson DL, Rice LB, Stelling J, Struelens MJ, Vatopoulos A, Weber JT, Monnet DL. 2012. Multidrug-resistant, extensively drug-resistant and pandrug-resistant bacteria: an international expert proposal for interim standard definitions for acquired resistance. Clin Microbiol Infect 18:268–281. doi:10.1111/j.1469-0691.2011.03570.x. 21793988

[12] Bevan ER, Jones AM, Hawkey PM. 2017. Global epidemiology of CTX-M β-lactamases: temporal and geographical shifts in genotype. J Antimicrob Chemother 72:2145–2155. doi:10.1093/jac/dkx146. 28541467

[13] Bush K. 2013. Proliferation and significance of clinically relevant β-lactamases. Ann N Y Acad Sci 1277:84–90. doi:10.1111/nyas.12023. 23346859

[14] Goldberg DW, Fernandes MR, Sellera FP, Costa DGC, Loureiro Bracarense AP, Lincopan N. 2019. Genetic background of CTX-M-15-producing *Enterobacter hormaechei* ST114 and *Citrobacter freundii* ST265 co-infecting a free-living green turtle (*Chelonia mydas*). Zoonoses Public Health 66:540–545. doi:10.1111/zph.12572. 30843359

[15] Igbinosa EO, Rathje J, Habermann D, Brinks E, Cho GS, Franz CMAP. 2018. Draft genome sequence of multidrug-resistant strain *Citrobacter portucalensis* MBTC-1222, isolated from uziza (*Piper guineense*) leaves in Nigeria. Genome Announc 6:e00123-18. doi:10.1128/genomeA.00123-18. 29496838 PMC5834338

[16] Loncaric I, Misic D, Szostak MP, Künzel F, Schäfer-Somi S, Spergser J. 2020. Broad-spectrum cephalosporin-resistant and/or fluoroquinolone-resistant Enterobacterales associated with canine and feline urogenital infections. Antibiotics 9:387. doi:10.3390/antibiotics9070387. 32645942 PMC7399855

[17] Hasan MS, Sultana M, Hossain MA. 2019. Complete genome arrangement revealed the emergence of a poultry origin superbug *Citrobacter portucalensis* strain NR-12. J Glob Antimicrob Resist 18:126–129. doi:10.1016/j.jgar.2019.05.031. 31185330

[18] Tsypin LM, Saunders SH, Bar-On Y, Leadbetter JR, Newman DK. 2020. Draft genome sequence of the redox-active enteric bacterium *Citrobacter portucalensis* strain MBL. Microbiol Resour Announc 9:e00695-20. doi:10.1128/MRA.00695-20. 32763937 PMC7409854

[19] Chang H, Mishra R, Cen C, Tang Y, Ma C, Wasti S, Wang Y, Ou Q, Chen K, Zhang J. 2021. Metagenomic analyses expand bacterial and functional profiling biomarkers for colorectal cancer in a Hainan cohort, China. Curr Microbiol 78:705–712. doi:10.1007/s00284-020-02299-3. 33410957

[20] Cao X, Xie H, Huang D, Zhou W, Liu Y, Shen H, Zhou K. 2021. Detection of a clinical carbapenem-resistant *Citrobacter portucalensis* strain and the dissemination of *Citrobacter portucalensis* in clinical settings. J Glob Antimicrob Resist 27:79–81. doi:10.1016/j.jgar.2021.04.027. 34048980

[21] Al-Bahry SN, Mahmoud IY, Al-Zadjali M, Elshafie A, Al-Harthy A, Al-Alawi W. 2011. Antibiotic resistant bacteria as bio-indicator of polluted effluent in the green t*urtles, Cheloniamydas* in Oman. Mar Environ Res 71:139–144. doi:10.1016/j.marenvres.2010.12.005. 21237506

[22] Sellera FP. 2019. Epidemiological implications of drug-resistant bacteria in wildlife rehabilitation centers. J Infect Public Health 12:748–749. doi:10.1016/j.jiph.2019.06.002. 31230952

